# A Prospective Study of Cutaneous Adverse Effects Among Patients With Topical Corticosteroid Abuse in a Tertiary Care Center

**DOI:** 10.7759/cureus.114068

**Published:** 2026-08-06

**Authors:** Annie Priya Dharshini Inbamani, Gayathri Sundaram, Rajalakshmi Ramalingam

**Affiliations:** 1 Dermatology, Srinivasan Medical College and Hospital, Dhanalakshmi Srinivasan University, Trichy, IND; 2 Dermatology, Srinivasan Medical College and Hospital, Trichy, IND

**Keywords:** awareness, cutaneous adverse effects, over the counter medication, over-the-counter medication, steroid abuse, topical corticosteroids

## Abstract

Background

Topical corticosteroids are widely used in dermatology because of their rapid anti-inflammatory effects. However, their easy availability and lack of awareness regarding their adverse effects have led to widespread misuse, resulting in a growing burden of steroid-induced cutaneous complications.

Objectives

This study aims to assess topical corticosteroid abuse, analyze the patterns and duration of use, identify the commonly used topical corticosteroid agents, and document the spectrum of associated cutaneous adverse effects.

Methods

A prospective observational study was conducted in the Department of Dermatology at Srinivasan Medical College & Hospital, Dhanalakshmi Srinivasan University, Trichy, Tamil Nadu, between November 2023 and December 2024. Adult patients with a history of topical corticosteroid use presenting with clinically evident corticosteroid-induced cutaneous adverse effects were consecutively enrolled after providing informed consent. Demographic data, indication for use, source of procurement, duration of application, and type of topical corticosteroid used were recorded. Patients underwent a detailed dermatological examination, and adverse effects were documented. Data were analyzed using descriptive statistics.

Results

A total of 120 patients were included, with a mean age of 33.88 ± 13.50 years. Females constituted the majority (60.8%, n=73). Non-prescription use of topical corticosteroids was observed in 60% (n=72) of patients. The most common indication for use was dermatophytosis (58.3%, n=70), followed by melasma (10%, n=12) and acne (5.8%, n=7). The duration of misuse ranged from less than one month to more than two years, with 31.7% of patients using topical corticosteroids for 1-3 months. Tinea incognito (33.3%, n=40) was the most frequent adverse effect, followed by acneiform eruptions (15%, n=18), acne flare (15.8%, n=19), and pigmentary changes.

Conclusion

Topical corticosteroid abuse is common and is largely driven by non-prescription use, particularly for fungal infections. The resulting cutaneous adverse effects cause significant morbidity. Strengthening regulatory control, promoting rational prescribing, and improving public awareness are essential to curb this preventable problem.

## Introduction

Topical corticosteroids are among the most commonly prescribed medications in dermatology because of their potent anti-inflammatory, immunosuppressive, antiproliferative, and vasoconstrictive properties. They are highly effective and safe when used appropriately, with the correct indication, potency, duration, and under the supervision of a medical professional [1]. However, their rapid symptomatic relief, easy availability, and lack of public awareness regarding their adverse effects have led to widespread misuse and abuse, particularly in developing countries such as India [2].

The misuse of topical corticosteroids has become a major public health concern in recent years. Over-the-counter availability, irrational fixed-dose combinations, and inadequate counseling by non-medical personnel such as pharmacists, beauticians, and friends have all contributed to their indiscriminate use [3]. Topical corticosteroids are commonly misused for conditions such as dermatophytosis, acne, melasma, skin lightening, and nonspecific pruritus, where their use is either contraindicated or requires strict medical supervision [4,5].

Prolonged and unsupervised use of topical corticosteroids may result in a wide range of cutaneous adverse effects, including tinea incognito, steroid-induced acne, perioral dermatitis, telangiectasia, striae, skin atrophy, pigmentary changes, and contact dermatitis [6,7]. The face and intertriginous areas are particularly susceptible because of increased percutaneous absorption, resulting in cosmetically disfiguring and psychologically distressing complications [8]. The rising incidence of steroid-modified dermatophytosis further highlights the inappropriate use of topical corticosteroids in fungal infections [9].

Despite the growing importance of this issue, data from tertiary care centers describing the demographic profile of affected patients, patterns and duration of misuse, and the spectrum of associated cutaneous adverse effects remain limited. Such information is essential for early identification of the problem, patient education, and the development of effective regulatory and preventive strategies [10]. Therefore, the present prospective study was conducted at a tertiary care center to evaluate the clinical profile of patients with topical corticosteroid abuse, analyze the patterns and duration of misuse, identify the commonly used topical corticosteroids, and document the associated cutaneous adverse effects.

## Materials and methods

This prospective observational study was conducted at the Department of Dermatology, Srinivasan Medical College & Hospital, Dhanalakshmi Srinivasan University, Trichy, Tamil Nadu, from November 2023 to December 2024, after obtaining approval from the Institutional Ethics Committee. A total of 120 consecutive patients attending the dermatology outpatient department with a history of topical corticosteroid application and clinically evident corticosteroid-induced cutaneous adverse effects were enrolled after providing written informed consent. Patients who had received only non-steroidal topical therapy, were on systemic corticosteroids, were pregnant or lactating, or declined to participate were excluded from the study.

Demographic details, including age, sex, place of residence, and educational status, were recorded using a predesigned structured proforma. Information regarding topical corticosteroid use, including the indication, source of procurement (prescription or non-prescription), duration and frequency of application, and the type of topical corticosteroid used, was documented. All participants underwent a comprehensive dermatological examination performed by a qualified dermatologist, and the cutaneous adverse effects were clinically identified and categorized. Additional investigations, such as potassium hydroxide (KOH) mount, were performed whenever clinically indicated to exclude fungal infections.

The primary outcome measure was the spectrum and frequency of cutaneous adverse effects associated with topical corticosteroid abuse. Secondary outcome measures included the patterns and duration of abuse and the types of topical corticosteroids most commonly used. Data management was performed using Microsoft Excel (Microsoft Corporation, Redmond, Washington), and statistical analysis was carried out with IBM SPSS Statistics for Windows, Version 26 (Released 2019; IBM Corp., Armonk, New York). Continuous variables were expressed as mean ± standard deviation, while categorical variables were summarized as frequencies and percentages.

## Results

Table 1 presents the demographic characteristics of the study participants.

**Table 1:** Demographic characteristics of patients

Parameters		Number of Patients (%)
Age (years)		33.88 ± 13.50
Gender	Male	47 (39.2%)
	Female	73 (60.8%)
Residence	Rural	74 (61.7%)
	Urban	46 (38.3%)
Education	Illiterate	7 (5.8%)
	Primary	17 (14.2%)
	Secondary	36 (30%)
	Graduate	44 (36.7%)
	Postgraduate	16 (13.3%)

A total of 120 patients were included in the study, with a mean age of 33.88 ± 13.50 years. Females constituted the majority of the study population (60.8%, n=73), while males accounted for 39.2% (n=47). Most participants were from rural areas (61.7%, n=74), whereas 38.3% (n=46) resided in urban areas. Regarding educational status, the largest proportion of patients were graduates (36.7%, n=44), followed by those with secondary education (30.0%, n=36), primary education (14.2%, n=17), and postgraduate education (13.3%, n=16). Illiteracy was observed in 5.8% (n=7) of the study population. The pattern of topical corticosteroid use and abuse is presented in Table 2.

**Table 2:** Pattern of steroid use and abuse

Variables		Number of Patients (%)
Source of corticosteroid	Prescription	48 (40%)
	Non-prescription	72 (60%)
Duration of corticosteroid abuse	<1 month	19 (15.8%)
	1–3 months	38 (31.7%)
	3–6 months	19 (15.8%)
	6 months–1 year	30 (25%)
	1–2 years	12 (10%)
	>2 years	2 (1.7%)

**Figure 1:**
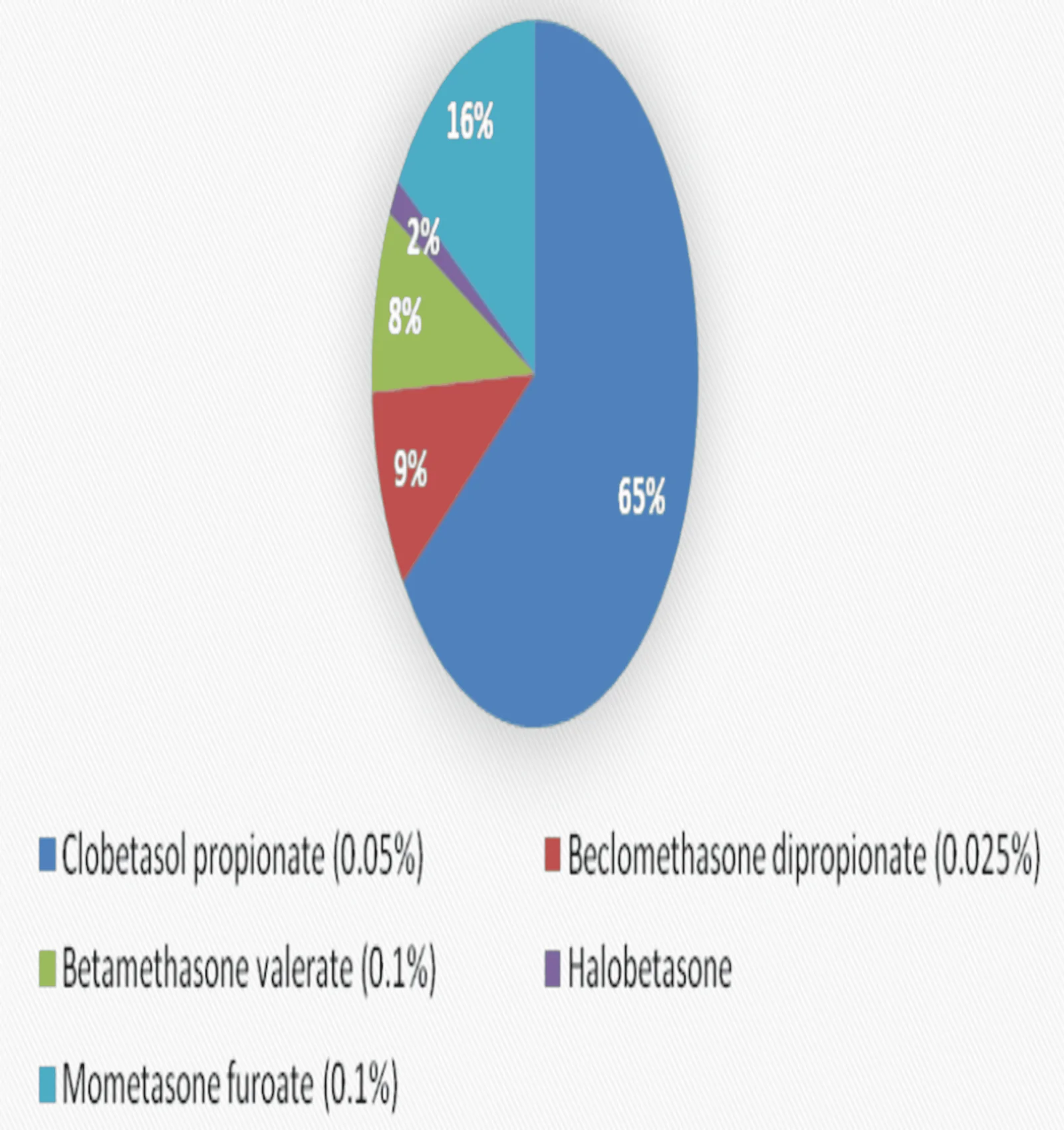
Distribution of topical corticosteroid agents used by study participants

Non-prescription use of topical corticosteroids was more common, accounting for 60.0% (n=72) of cases, whereas prescription-based use was reported by 40.0% (n=48) of patients. Regarding the duration of abuse, the highest proportion of patients had used topical corticosteroids for 1-3 months (31.7%, n=38), followed by 6 months to 1 year (25.0%, n=30). Use for less than 1 month and for 3-6 months was observed in 15.8% (n=19) of patients each. Prolonged abuse for more than 1 year was reported by 10.0% (n=12) of patients, while only 1.7% (n=2) had used topical corticosteroids for more than 2 years. Figure 1 shows the distribution of the commonly used topical corticosteroid agents.

Clobetasol propionate (0.05%) was the most commonly used topical corticosteroid, accounting for 65% of the cases. This was followed by mometasone furoate (0.1%) (16%), beclomethasone dipropionate (0.025%) (9%), and betamethasone valerate (0.1%) (8%). Halobetasol propionate was the least commonly used topical corticosteroid, accounting for only 2% of cases. Overall, the findings indicate the predominant misuse of high-potency topical corticosteroids. Topical corticosteroid formulations misused among the study participants are shown in Table 3. The indications for topical corticosteroid use are presented in Table 4.

**Table 3:** Topical corticosteroid formulations misused among the study participants (n = 120)

Topical corticosteroid formulation	Number of patients (n)
Clobetasol propionate (0.05%)	78
Betamethasone valerate (0.1%)	18
Beclomethasone dipropionate (0.025%)	11
Mometasone furoate (0.1%)	10
Multiple corticosteroid formulations	3
Total	120

**Table 4:** Clinical indications for topical corticosteroid use among patients

Indication	Number of patients (%)
Dermatophytosis	70 (58.3%)
Acne	7 (5.8%)
Melasma	12 (10%)
Skin lightening	5 (4.2%)
Oily skin	5 (4.2%)
Itching	9 (7.5%)
Facial dryness	1 (0.8%)
Others	11 (9.2%)

**Figure 2:**
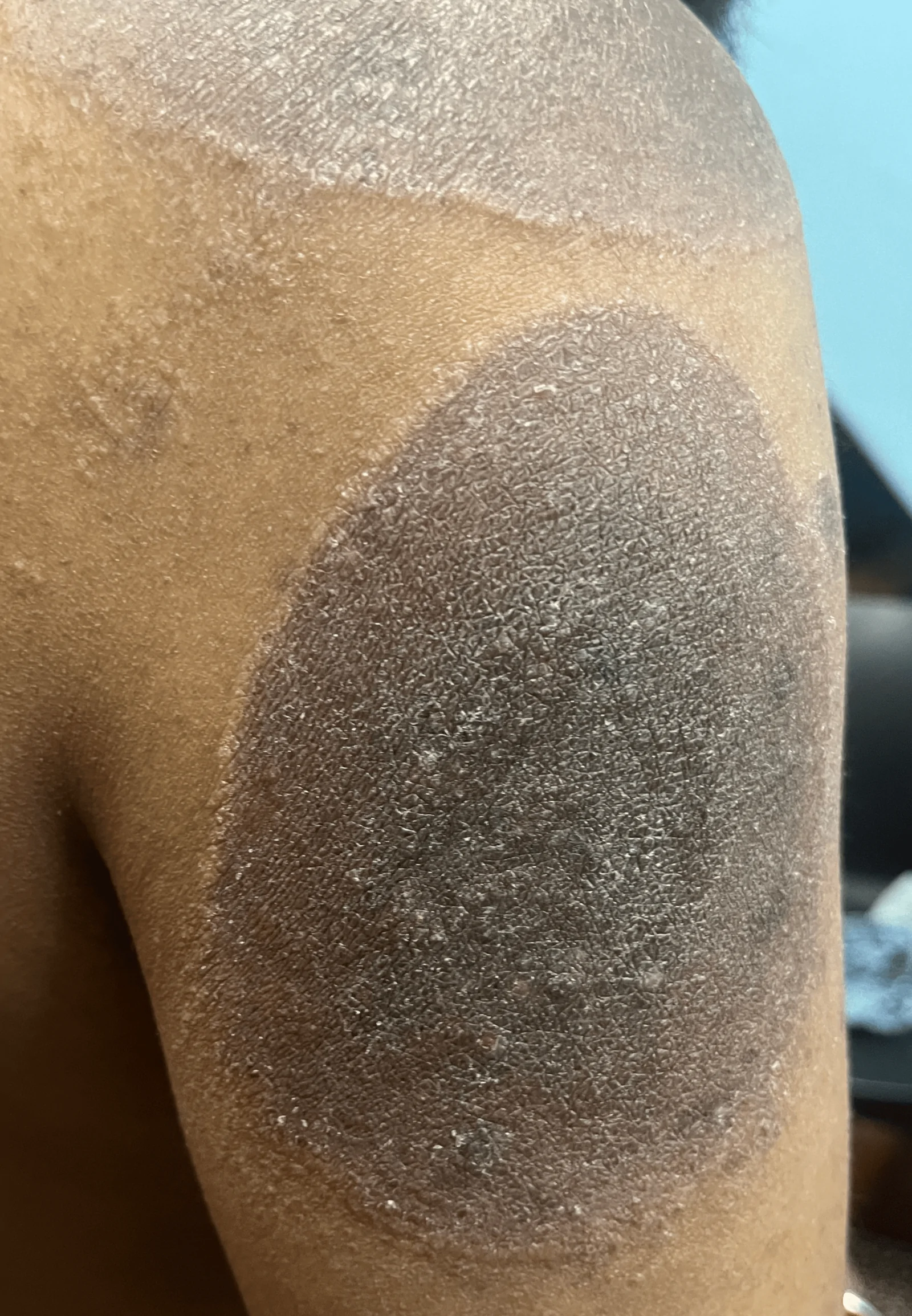
Tinea incognito

**Figure 3:**
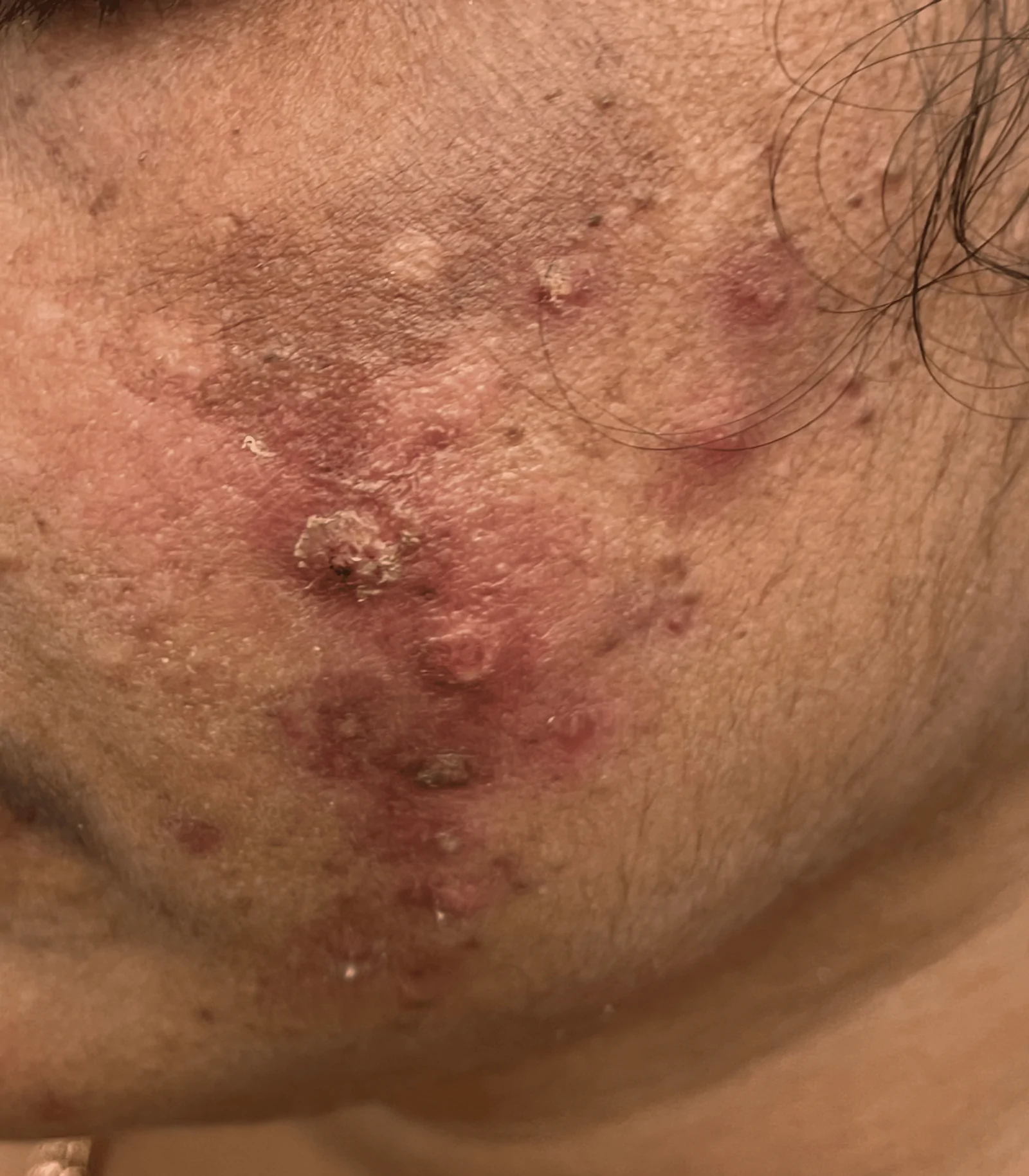
Melasma with steroid-induced acneiform eruptions

**Figure 4:**
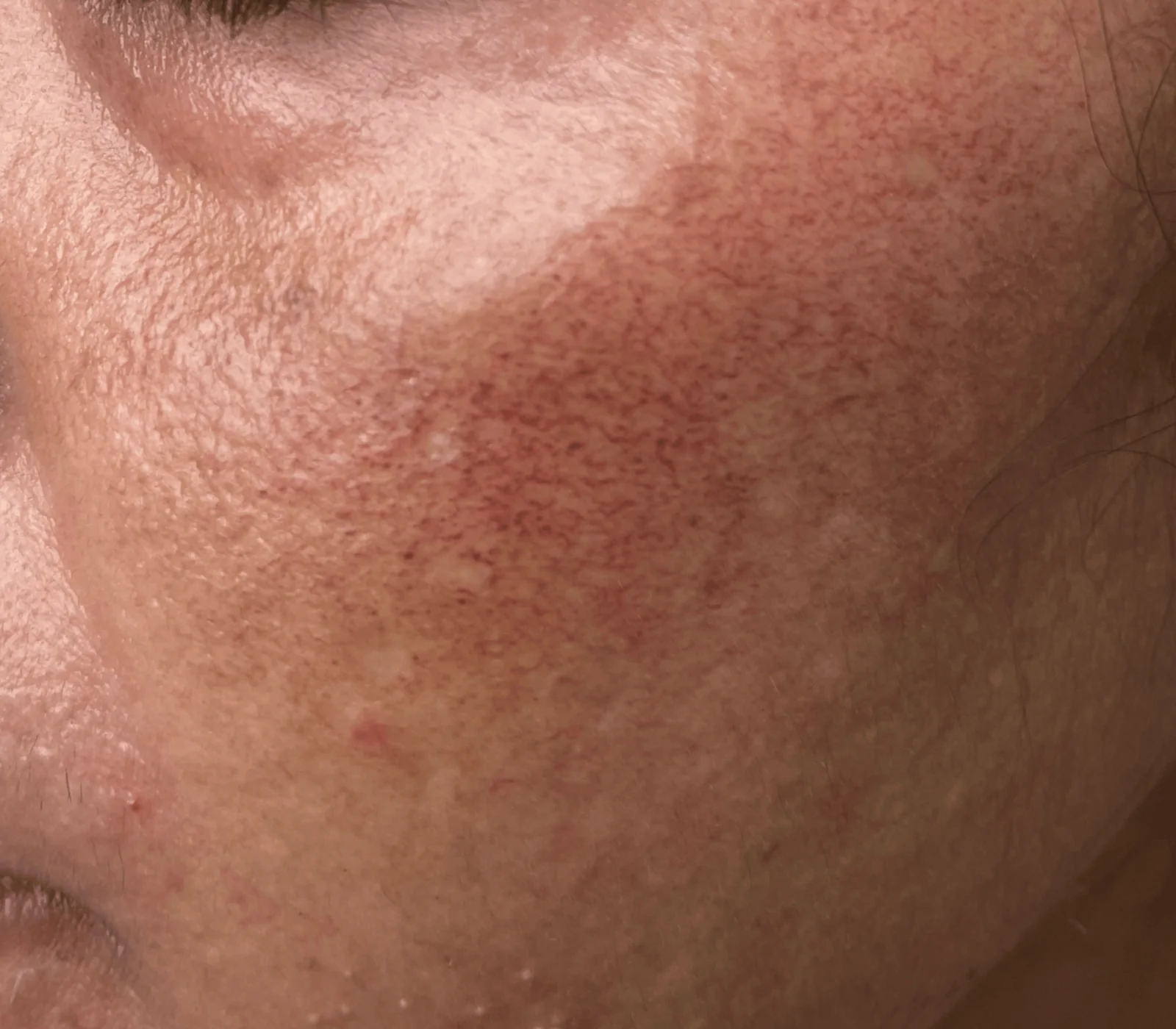
Telangiectasia

**Figure 5:**
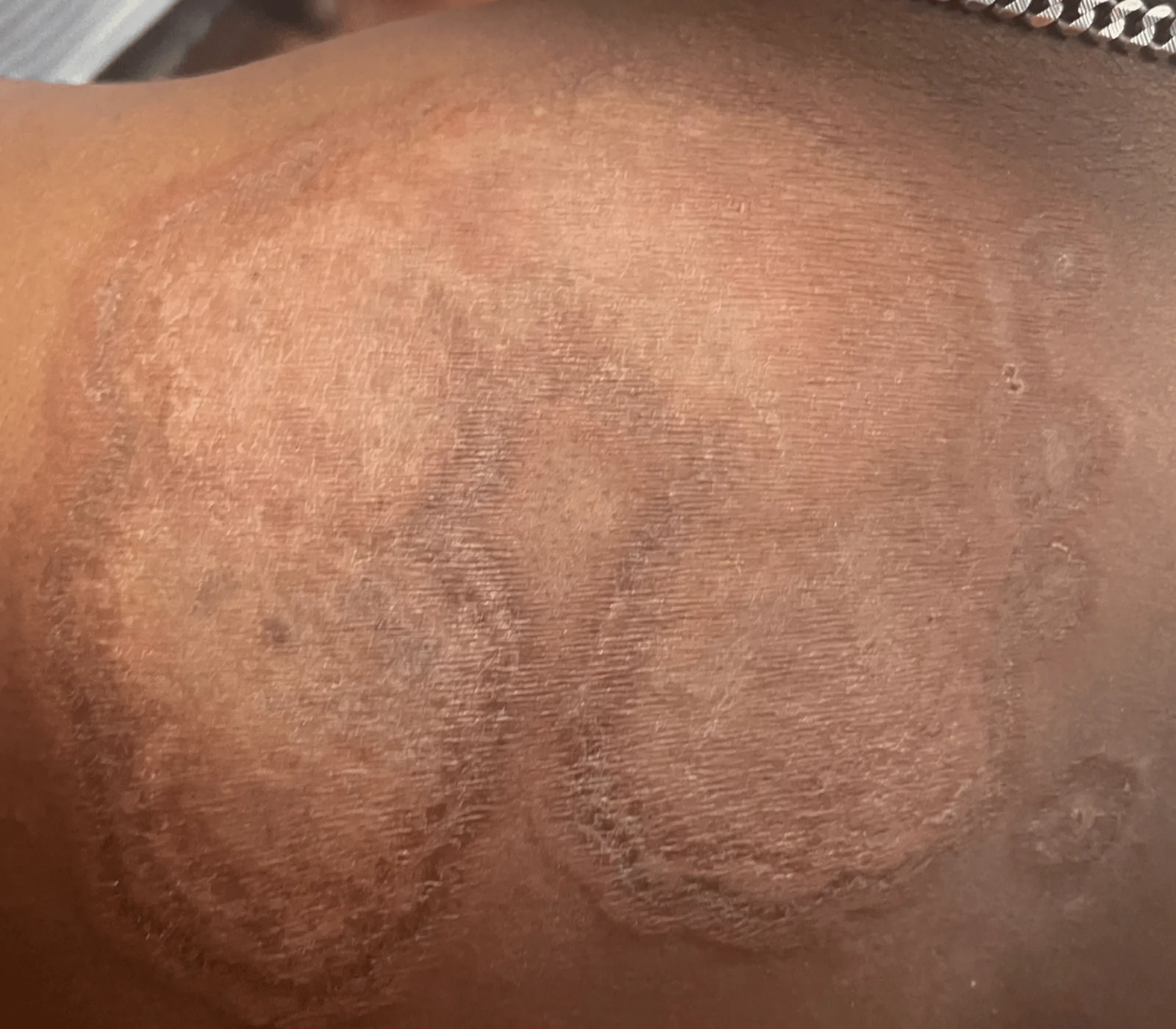
Steroid-induced tinea incognito with post-inflammatory hypopigmentation

**Figure 6:**
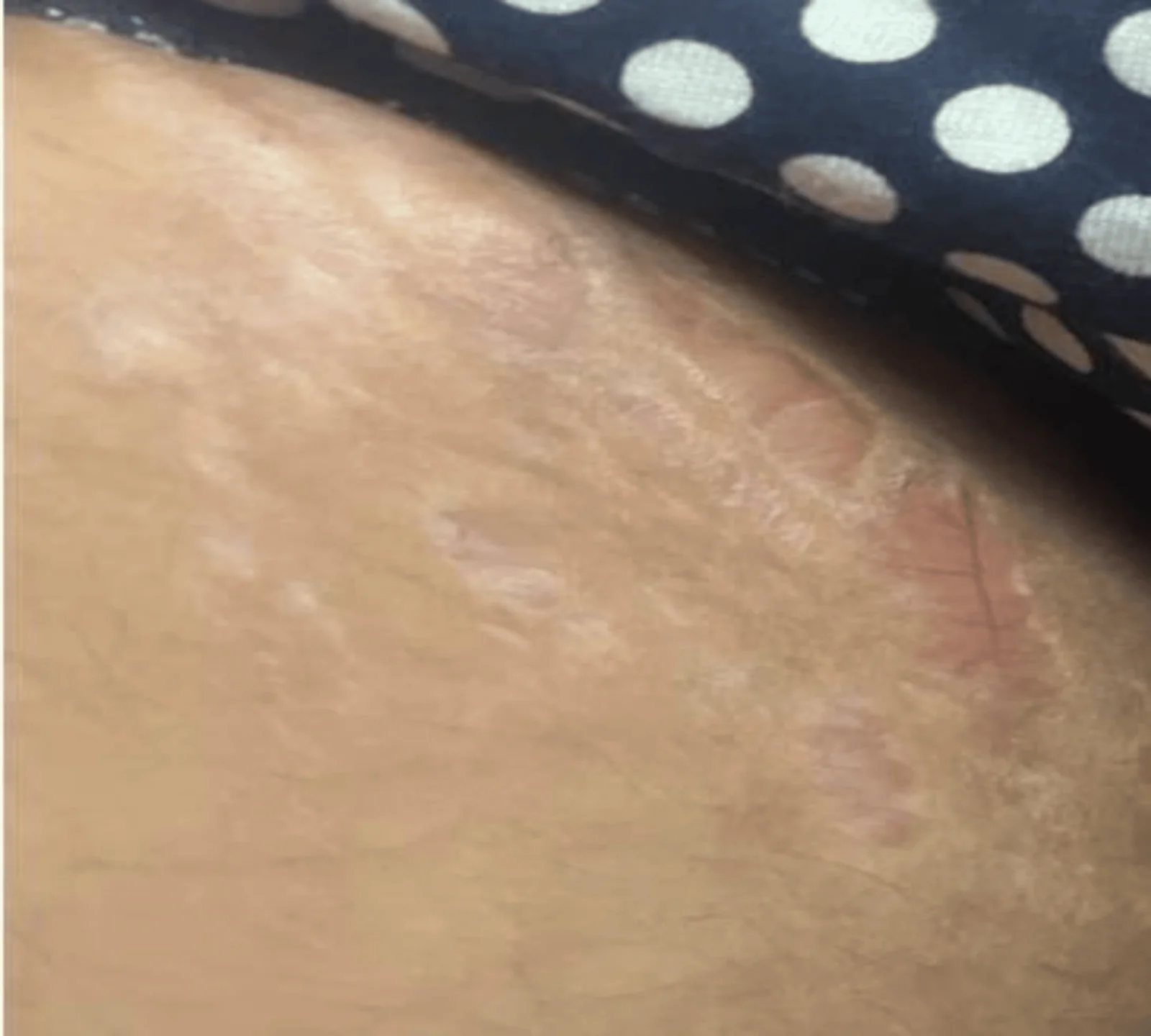
Steroid-induced striae

 Dermatophytosis was the most common indication for topical corticosteroid use, reported by 58.3% (n=70) of patients. This was followed by melasma in 10.0% (n=12), itching in 7.5% (n=9), and acne in 5.8% (n=7) of patients. Cosmetic indications, including skin lightening and oily skin, were each reported by 4.2% (n=5) of patients. Facial dryness accounted for 0.8% (n=1), while other indications comprised 9.2% (n=11) of cases. Representative clinical photographs are shown in Figures 2-6.

The cutaneous adverse effects observed following topical corticosteroid use are presented in Table 5.

**Table 5:** Adverse effects observed following topical corticosteroid use

Adverse effects	Number of patients (%)
Tinea incognito	40 (33.3%)
Steroid-induced acneiform eruptions	18 (15%)
Acne flare	19 (15.8%)
Telangiectasia	4 (3.3%)
Striae	5 (4.2%)
Skin atrophy	1 (0.8%)
Hypopigmentation	19 (15.8%)
Perioral dermatitis	1 (0.8%)
Hyperpigmentation	5 (4.2%)
Contact dermatitis	4 (3.3%)
Others	4 (3.3%)

Tinea incognito was the most common adverse effect following topical corticosteroid use, affecting 33.3% (n=40) of patients. Acne-related adverse effects were also common, with acne flare observed in 15.8% (n=19) of patients and steroid-induced acneiform eruptions in 15.0% (n=18) of patients. Hypopigmentation was reported in 15.8% (n=19) of cases. Less common adverse effects included striae and hyperpigmentation (4.2%, n=5), telangiectasia and contact dermatitis (3.3%, n=4), and skin atrophy and perioral dermatitis (0.8%, n=1). Other miscellaneous adverse effects accounted for 3.3% (n=4) of cases.

## Discussion

Topical corticosteroids are widely used in dermatological practice because of their potent anti-inflammatory and immunosuppressive properties. However, their misuse and overuse have emerged as a major public health concern, particularly in developing countries where these medications are readily available over the counter and are frequently marketed as irrational fixed-dose combinations. The present prospective study highlights the pattern of topical corticosteroid abuse and the spectrum of associated cutaneous adverse effects in patients attending a tertiary care center.

The present study found that 60.0% of patients used topical corticosteroids without a prescription, indicating their easy availability and inadequate regulatory control. Similar findings were reported by Saraswat et al., who observed non-prescription use in 59.3% of patients [3]. Likewise, Meena et al. reported that more than half of their study population had used topical corticosteroids without a prescription, highlighting the widespread practice of unsupervised steroid use in the community [11]. The involvement of pharmacists, non-dermatologist prescribers, and self-medication has been consistently identified as major factors contributing to topical corticosteroid misuse [2].

Clobetasol propionate (0.05%), a superpotent topical corticosteroid, was the most frequently abused agent in the present study, accounting for 65.0% of cases. Saraswat et al. similarly reported that 44.3% of patients used clobetasol propionate (0.05%), which is comparable to our findings [3]. Another study by Dey VK reported that 88.92% of patients used potent or superpotent topical corticosteroids, either alone or in combination with antibiotics and/or antifungal agents, further emphasizing the widespread misuse of high-potency corticosteroids [5].

Dermatophytosis was the most common indication for topical corticosteroid misuse in the present study. The inappropriate use of topical corticosteroids in fungal infections suppresses local immunity, alters the clinical appearance of lesions, masks the classical features of infection, and leads to the development of tinea incognito. Accordingly, tinea incognito was the most common adverse effect observed in our study, affecting 33.3% of patients. Similar findings have been reported by Verma et al. and Panda et al., who identified topical corticosteroid misuse as a major contributor to the increasing burden of chronic and steroid-modified dermatophytosis in India [4,12]. Meena et al. also reported tinea incognito (49.46%) and steroid-induced acne (30.27%) as the most common adverse effects associated with topical corticosteroid misuse [11].

The duration of topical corticosteroid abuse varied considerably among the study participants, with many patients using these agents continuously for several months. Prolonged use of potent topical corticosteroids increases the risk of irreversible adverse effects such as striae, telangiectasia, and skin atrophy [13]. Although these complications were less frequent in our study, they remain clinically significant because of their permanent nature. Similar observations were reported by Coondoo et al., who demonstrated that the risk and severity of adverse effects increase with the potency of the corticosteroid, duration of application, and use under occlusion, which is consistent with the findings of the present study [2].

This study was conducted at a single tertiary care center, which may limit the generalizability of the findings to the wider population. The duration and pattern of topical corticosteroid use were based on patient-reported history and were therefore subject to recall bias. Furthermore, the absence of a control group limited the comparative assessment of risk factors associated with topical corticosteroid abuse.

## Conclusions

This prospective study highlights the substantial burden of cutaneous adverse effects associated with topical corticosteroid abuse in a tertiary care setting. Potent topical corticosteroids, particularly clobetasol propionate, were frequently misused without medical supervision and applied for prolonged periods and inappropriate indications. The majority of patients used topical corticosteroids for dermatophytic infections and cosmetic purposes, resulting in a high prevalence of steroid-modified dermatoses, including tinea incognito, acneiform eruptions, pigmentary changes, and other steroid-induced complications. These findings underscore the role of inadequate public awareness, unrestricted over-the-counter availability, and insufficient regulatory oversight in perpetuating topical corticosteroid abuse. Strengthening public education, enforcing prescription-only dispensing, and promoting rational prescribing practices are essential to curb misuse and reduce the burden of steroid-related dermatological morbidity.
